# Role of Mitochondria in Environmentally and Dietary Modulated Age-Associated Diseases

**DOI:** 10.3390/cells12030404

**Published:** 2023-01-25

**Authors:** Natascia Ventura, Judith Haendeler

**Affiliations:** 1 Institute of Clinical Chemistry and Laboratory Diagnostics, Medical Faculty, University of Duesseldorf, 40225 Duesseldorf, Germany; 2 The IUF-Leibniz Research Institute for Environmental Medicine, 40225 Duesseldorf, Germany; 3 Environmentally-Induced Cardiovascular Degeneration, Institute of Clinical Chemistry and Laboratory Diagnostics, Medical Faculty, University of Duesseldorf, 40225 Duesseldorf, Germany

Aging is an intricate and unavoidable phenomenon characterized by progressive accumulation of damage to cellular structural components with consequent decline in physiological functions and development of different pathological conditions, which lead to increase in frailty and mortality risk and bring a huge economic burden in our society. Aging is considered the number one risk factor for the development and progression of many chronic disorders ranging from cardiovascular diseases and diabetes to different types of cancer and neurodegenerative disorders. Genetic and environmental factors concurrently shape aging and age-associated diseases. A variety of genetically regulated mechanisms affecting aging have been identified in the past 50 years, also as a result of the exploitation of multicellular genetically tractable model organisms. A growing body of evidence indicates that environmental factors, including diet, impact human health and aging. On the one hand, external factors such as pesticides, radiation or dietary components as well as products of endogenous metabolism may negatively impact disease development and progression and may accelerate the aging process. On the other hand, several food components and supplements have been described or proposed to promote health and longevity. Nonetheless, these observations originate primarily from epidemiological or correlative studies, and while some molecular mechanisms have been involved in the modulation of cell and organism homeostasis in response to a wide range of external toxicants and insults, more mechanistic investigations are necessary to establish beneficial causal effect role of dietary interventions.

A number of aging theories have been proposed, and the “mitochondrial free radical theory of aging” (MFRTA) has taken center stage in this area of research for several decades. According to this theory, reactive oxygen species (ROS) generated by the mitochondrial respiratory chain (MRC) are the main cause of molecular damages. This theory has been questioned in recent years by the growing body of evidence showing mild increase in ROS and mild suppression of mitochondrial activity can actually promote health and lifespan across species. Nonetheless, there is no doubt that mitochondria play a pivotal role in cell homeostasis, e.g., through the regulation of energy metabolism, cellular redox status and cell death pathways. Accordingly, a wide spectrum of structural and functional mitochondria alterations, as well as the imbalance redox status, are characteristic features of the aging process and play an important role in the pathogenesis of different age-associated diseases. Environmental factors such as pesticides, radiation, cigarette smoke, and exposure to metals may also exert mitochondrial toxicity in different ways, e.g., by altering mtDNA integrity, inhibiting MRC complexes, or activating pro-apoptotic signaling. Moreover, as mitochondria are key players of cellular energy metabolism, they also represent central hubs in sensing and usage of dietary factors.

With this Special Issue, we aimed to highlight some of the current knowledge on the important role of mitochondria in environmentally- and dietary-modulated aging and associated diseases.

In this context, the review by Bortoli, Coumal and colleagues provides a comprehensive summary of the literature describing the role of mitochondrial dysfunction as a hallmark of environmental injury [1]. The authors review the variety of mechanisms of action of pollutants causing mitochondrial toxicity especially linked to chronic diseases. Moreover, they propose the Aryl hydrocarbon Receptor (AhR) function as an “exposome receptor”, whose activation by environmental pollutants leads to various toxic events through mitochondrial dysfunction. Lastly, they provide some important remarks related to mitochondrial toxicity and risk assessment.

Altschmied, Haendeler and colleagues, in turn, present a neat review of the current knowledge on the protective effects of curcumin, a polyphenolic compound naturally found in the Curcuma longa (tumeric), on treatment of cardiovascular diseases, with a special focus on its impact on oxidative stress and mitochondria [2]. The authors first critically describe cardiovascular diseases along with their major modifiable (obesity) and non-modifiable (aging) risk factors. Then, they provide a comprehensive summary of the different beneficial effects of curcumin on cellular senescence and age-related cardiovascular dysfunction, adipose tissue and obesity, atherosclerosis and myocardial infarction. In this context, they summarize curcumin molecular mechanisms related to mitochondria function and cellular redox homeostasis and indicate future directions of research, i.e., methods to improve compound bioavailability and better standardize clinical trials, necessary to fully exploit the beneficial health properties of this promising nutraceutical.

Two additional reviews describe very important mitochondrial functions, namely one-carbon metabolism and mitochondrial protein import in the context of aging and age-associated diseases. Tavernarakis and colleagues [3] first provide a comprehensive summary of cytosolic, nuclear and mitochondrial one-carbon metabolism, a network of interconnected biochemical reactions delivering one-carbon units to various biosynthetic pathways, and point out the ways in which dietary intake of micronutrients contributes to this metabolic network, thereby adapting the cellular metabolic state to environmental inputs. Furthermore, they specifically recapitulate the impact of some of these one-carbon metabolism-regulated pathways (i.e., folate, methionine, homocystein, transsulfuration and gluthatione homeostasis) on aging and neurodegeneration across species, with a particular focus on Alzheimer and Parkinson diseases.

The review from Borshchevskiy and colleagues [4] present an appealing summary of the state-of-the-art knowledge about the mitochondrial import machinery, an extensive network of specialized proteins required for correct import and sorting of more than 1500 nuclear-encoded proteins into their designated mitochondrial compartments. Moreover, they describe the mechanistic and casual involvement of defects in this fundamental system in age-related neurodegenerative (Alzheimer and Parkinson) as well as cardiovascular diseases, highlighting current research gaps in the field.

Finally, the original research work of Ventura and colleagues [5] exploits the nematode *C. elegans*, a powerful genetically tractable model organisms for aging and intervention studies, for an in vivo phenotype-based screening in search of interventions acting through mitochondria with beneficial effects on healthspan. To this aim, the authors exploit a high-content microscopy platform to search for natural compounds able to induce phenotypes (i.e., reduced animals’ size and induction of mitochondrial stress response genes) associated with mild mitochondrial stress, which is known to promote animal health- and lifespan across species. The screen led to the identification of four compounds i.e., isobavachalcone, manzamine A, kahalalide F and lutein consistently affecting different nematode phenotypes, which were subsequently validated in Drosophila cells for their effects on mitochondria activity and lipid content. Out of the four, kahalalide F and lutein were chosen for further age-related analysis, and were shown to promote resistance to stress, health- and lifespan. Interestingly, initial mechanistic investigation reveals that the *C. elegans* ortholog of the synaptic regulatory protein neuroligins, nlg-1, was induced by the two compounds and mediated lutein healthspan effects.

Overall, the five papers offer an interesting overview of the possible opposite impact that environmental and dietary factors can have on aging and associated diseases via modulation of different mitochondrial-associated activities and metabolic pathways, the former in most cases acting as mitochondrial toxicants while the latter functioning as beneficial agents or nutraceuticals.
